# The Pfam protein families database in 2019

**DOI:** 10.1093/nar/gky995

**Published:** 2018-10-24

**Authors:** Sara El-Gebali, Jaina Mistry, Alex Bateman, Sean R Eddy, Aurélien Luciani, Simon C Potter, Matloob Qureshi, Lorna J Richardson, Gustavo A Salazar, Alfredo Smart, Erik L L Sonnhammer, Layla Hirsh, Lisanna Paladin, Damiano Piovesan, Silvio C E Tosatto, Robert D Finn

**Affiliations:** 1 European Molecular Biology Laboratory, European Bioinformatics Institute (EMBL-EBI), Wellcome Trust Genome Campus, Hinxton, Cambridge CB10 1SD, UK; 2 HHMI, Harvard University, 16 Divinity Ave Cambridge, MA 02138 USA; 3 Science for Life Laboratory, Department of Biochemistry and Biophysics, Stockholm University, 17121 Solna, Sweden; 4 Department of Biomedical Sciences, University of Padua, 35131 Padova, Italy; 5 Dept. of Engineering, Pontificia Universidad Católica del Perú 1801, San Miguel 15088, Lima, Perú

## Abstract

The last few years have witnessed significant changes in Pfam (https://pfam.xfam.org). The number of families has grown substantially to a total of 17,929 in release 32.0. New additions have been coupled with efforts to improve existing families, including refinement of domain boundaries, their classification into Pfam clans, as well as their functional annotation. We recently began to collaborate with the RepeatsDB resource to improve the definition of tandem repeat families within Pfam. We carried out a significant comparison to the structural classification database, namely the Evolutionary Classification of Protein Domains (ECOD) that led to the creation of 825 new families based on their set of uncharacterized families (EUFs). Furthermore, we also connected Pfam entries to the Sequence Ontology (SO) through mapping of the Pfam type definitions to SO terms. Since Pfam has many community contributors, we recently enabled the linking between authorship of all Pfam entries with the corresponding authors’ ORCID identifiers. This effectively permits authors to claim credit for their Pfam curation and link them to their ORCID record.

## INTRODUCTION

Pfam is a database of protein families (1,2). Briefly, each Pfam database entry is comprised of a seed alignment, which forms the basis to build a profile hidden Markov model (HMM) using the HMMER software (http://hmmer.org/) (3,4). The profile HMM is then queried against a sequence database called *pfamseq*, and all matches scoring above the curated threshold (carefully chosen to avoid the inclusion of any known false positives), are aligned back to the profile HMM to generate the full alignment. Where possible, each entry is annotated with functional information derived from literature. To improve sustainability, especially with regard to scaling of the resource, *pfamseq* is derived only from the UniProt Knowledgebase (UniProtKB) (5) sequences that belong to Reference Proteomes (2), rather than the entirety of UniProtKB. This data is available on our website (https://pfam.xfam.org) while our FTP site (ftp://ftp.ebi.ac.uk/pub/databases/Pfam/current_release) contains flatfiles, including exports of the MySQL database for current and other releases over the past decade.

Generally, Pfam aims to cover as much of protein sequences as possible with the fewest number of models (6). Typically, an individual entry is searched iteratively so as to incorporate distantly related sequences that are believed to have been derived from a common ancestral protein. However, although all sequences are related, they may not share the same function. For example, the Peptidase_M14 entry (Pfam: PF00246) contains both active and inactive homologues and can be subdivided into at least four subfamilies. Of these, carboxypeptidase A1 favours substrate residues in the cleavage site with aromatic or branched side chains, while carboxypeptidase E favours basic amino acids (7). Despite our efforts to make Pfam entries as comprehensive as possible, it is important to remember that no two Pfam entries are allowed to overlap, i.e. two families that match the same amino acid residue; see the full description in (2). However, some superfamilies such as the Rossmann fold are so diverse that a single profile HMM is insufficient to capture the entire diversity. To tackle such cases, Pfam entries that are known to be evolutionarily related are grouped together into Pfam Clans (8). For example, Pfam clan CL0063 encompasses 198 entries belonging to the FAD/NAD(P)-binding Rossmann fold superfamily. Two Pfam entries from the same clan may overlap, but a post-processing step on the HMMER search results resolves these overlaps, ensuring that only one family matches to a particular region of a protein.

Each entry is tagged with one of six different *types* in Pfam: *family, domain, motif, repeat, coiled coil* or *disordered*, indicating the class of the functional unit being represented by that entry. Types *family* and *domain* are the most common (6,248 and 11,177 entries, respectively), comprising over 97.2% of all entries. Type *domain* is usually distinguished from type *family* by a known structure that indicates that the entry represents a single globular domain. However, the context of surrounding Pfam entries can delineate a conserved ‘domain’ in some cases.

Although minor changes have been made to the Pfam website since the last publication (2), we have undertaken a number of efforts to improve the content as well as increase the breadth of Pfam entries. New users to Pfam are encouraged to follow the new Online Training ‘Quick Tour’ that offers a primer to using the resource (https://www.ebi.ac.uk/training/online/course/pfam-quick-tour).

Here, we describe the most recent release of Pfam (version 32.0) and provide details on the underlying work that has contributed to the release. These include small yet important flatfile format changes, as well as providing better credit for authors of Pfam entries (both new and updated ones), many of whom are members of the Pfam user community.

## Pfam VERSION 32.0

Pfam 32.0, which released in September 2018 contains a total of 17,929 entries. Of all the sequences in UniProtKB, 77.2% have at least one match to a Pfam entry, while 53.2% of all residues fall within a Pfam entry (termed sequence and residue coverage, respectively). The Pfam sequence and residue coverage of UniProtKB has remained fairly constant since Pfam 29.0 (released in 2015, wherein the corresponding UniProtKB sequence coverage was 76.1% and residue coverage was 54.8%) (2). UniProtKB concurrently increased by 65 million sequences, a growth of 128%. On the other hand, the residue coverage of the reference proteomes in *pfamseq* significantly increased since Pfam 29.0. The sequence and residue coverage of the reference proteomes in Pfam 32.0 is 74.5% (an increase of 1.0%) and 50.1% (an increase of 3.1%), respectively. Between Pfam releases 29.0 and 32.0, the reference proteomes sequence database, and hence *pfamseq*, increased in size by 34 million sequences (a growth of 283%).

These new sequences cover an ever broader range of taxonomies, with the UniProtKB redundancy procedures (9) ensuring that growth reflects increased diversity, rather than, for instance, additional strains of the same bacterial species. Thus, these coverage statistics have been maintained as a result of new sequences being matched by existing Pfam entries, and the generation of 1,664 new entries.

As mentioned previously, Pfam clans are a classification of Pfam entries that reflect their evolutionary relationships. We use both sequence and structural information to determine whether two Pfam entries should belong to the same clan. As the volume of sequence and structure data is constantly expanding, adding entries to clans is an ongoing activity that parallels the detection of new relationships. We aim to ensure that Pfam entries and clan relationships are consistent with other structural classifications [e.g. CATH (10), SCOP (11)], and entries are consistent with each other within a clan, ideally having the same Pfam type and models of comparable sizes. Unlike entries which are not members of clans, two Pfam entries belonging to the same clan are allowed to overlap, i.e. the models can match the same region on a sequence as described earlier. These overlaps are then removed during a post-processing step, ensuring that only the most significant match (lowest E-value) is retained. Sequence regions that are in the seed alignment for an entry constitute an exception, in which case these remain with that entry.

There are 628 clans in total within Pfam 32.0, with 74 new clans added since Pfam 29.0. There has been a concurrent effort to identify relationships between Pfam entries. The number of entries belonging to clans has grown from 5,282 in Pfam release 29.0 to 7,001 in release 32.0 reflecting an overall increase of 1,719 entries (note: a small number of Pfam entries have been removed, merged and/or deleted). Although this number represents less than 40% of entries in Pfam, it corresponds to 74% of all sequence regions annotated by Pfam (an increase of 6% since Pfam 29.0). A small number of relationships have been detected solely based on sequence data. For example, using the Simple Comparison Of Outputs Program (12), we performed a comprehensive “all-against-all” analysis that identified an additional 22 Pfam entries which were consequently added to a clan. However, the majority of the recent additional relationships between Pfam entries have been identified by comparison to the Evolutionary Classification of Protein Domains (ECOD) database (13).

### Improving the content of Pfam using ECOD

ECOD is a hierarchical classification of protein domains based on evolutionary relationships determined from known structures. Detailed comparisons between Pfam (version 31.0) and a subset of ECOD (version 29) have guided the inclusion of the majority of the new Pfam entries, improved family definitions and entries added to clans. The F-group level (family level) in ECOD is primarily derived from Pfam, i.e. based on sequence similarity. This allows us to readily compare Pfam entries to the F-group entries in ECOD and follow higher levels of classification within ECOD (H-groups). In other words, we can group similar Pfam entries into clans based on comparing the ECOD F-level grouping within the H-level. However, where no corresponding entry in Pfam is found, the ECOD resource generates an ECOD Unclassified Family (EUF) (14). As such, these EUFs represented a source of potential new Pfam entries and thus, were compared in detail.

To incorporate the EUFs, we performed a search of the corresponding profile HMMs from ECOD against *pfamseq*. Significant matches (those with E-values < 0.001) were aligned back to the profile HMM to construct a new seed alignment based on *pfamseq*. This new seed alignment was in turn used to construct a new profile HMM and then, searched as before. The resulting matches were then compared to Pfam to look for overlaps with existing entries and partitioned into two groups: (1) those that lacked any overlapping matches; and (2) those that contained one or more matches that overlapped with a pre-existing Pfam match. Those EUFs within group 1 were then subjected to curator-driven iterative searching as with any Pfam entry, to ensure that the family was as comprehensive as possible. This process resulted in some entries within group 1 overlapping with other EUFs within group 1, while few others overlapped with a Pfam entry and were subsequently moved to group 2; those that did not overlap after iteration were added to Pfam as new entries. Having exhausted this set, the overlaps with Pfam (group 2) were evaluated to understand the relationship with the EUFs.

**Figure 1.**
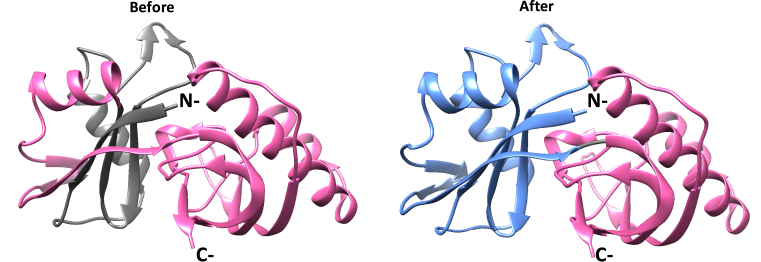
The modification of the domain structure of PDB:4z7k (UniProt: A4FXZ3) between Pfam releases 31.0 and 32.0. The structure of PDB:4z7k is represented as a ribbon cartoon of the c-α backbone, with PF17262 coloured in pink. Regions not covered by Pfam are coloured grey. The new Pfam entry PF17955 is coloured blue. In release 31.0 (left panel), the domain boundaries for PF17262 (pink, PDB residues 67–217) extend into the N-terminal structural domain. The coverage of the same structure by Pfam 32.0 (right panel). The C-terminal domain, PF17262 (PDB residues 107–218) boundaries have been corrected and renamed from DUF5328 to Cas6b_C. A new Pfam entry PF17955, named Cas6b_N was created (blue, PDB residues 1–105) to represent the N-terminal domain.

In those cases where the domain boundaries were in concordance with Pfam, the Pfam entry was iterated in an attempt to incorporate the additional matches represented by the EUF. Iterating Pfam entries can be non-trivial, as it is not always possible to improve the sensitivity of the model without affecting the specificity. If the EUF-based entry was sufficiently large, the overlaps were resolved, typically by adding the family into an existing clan or creating a new clan. In other cases, the iteration of the Pfam entry would result in overlaps with non-homologous entries (e.g. significant terminal overlaps) and were no longer considered. When the domains were incongruent, the overlapping Pfam domain would be modified to prevent the overlaps. For example, Pfam entry DUF5328 (a ‘domain of unknown function’ [DUF], Pfam: PF17262) from Pfam 31.0 was identified as corresponding to two ECOD domains. Based on the ECOD domain boundaries for the Protein Data Bank (PDB) (15,16) accession 4z7k (ECOD domain: e4z7kB2), we removed the N-terminal region of DUF5328 in line with the structural domain. This modified domain corresponded to the C-terminal domain of Cas6b proteins and was accordingly renamed to Cas6b_C (Pfam: PF17262), adding both functional annotation and literature references. Furthermore, the structural information prompted us to add it to the RAMPS-Cas5-like clan (Pfam: CL0362). Additionally, using the region of the structure N-terminal to PF17262 (ECOD family: EUF07099, version 29.0), we created a new Pfam entry called Cas6b_N (Pfam: PF17955). Cas6b_N adopts a similar structure to Cas6b_C and was therefore also added to clan CL0362. Figure 1 shows the change in the domain boundaries for PF17262 and the addition of the PF17955 entry between Pfam 31.0 and Pfam 32.0, on PDB structure PDB: 4z7k.

Due to the complex set of additions and changes to Pfam coupled with the multiplicity of EUFs added to new and existing entries, only a summary of the changes to Pfam are presented here. Overall, 825 new Pfam entries (50%) have been generated using ECOD, with over 400 existing entries changing their domain boundaries between Pfam releases 29.0 and 32.0. As indicated in Figure 1, this has helped us improve the consistency of Pfam domains with known structures and increase our coverage of them. As such, Pfam 32.0 now covers 87.1% of sequences and 73.8% of all residues represented by known structures found in PDB (15,16) (9 May 2018). Again, it is important to note that the marginal increase (<1%) in both these coverage metrics since Pfam 29.0 must be evaluated in the context of the corresponding growth of PDB by 37% (105,494 sequences).

### Improvements to Pfam type definitions

The Findable, Accessible, Interoperable and Reusable data principles [FAIR] (17) are an important set of guidelines for data resource providers. One approach to improving interoperability is the adoption of ontologies which allow, for instance, biological terms to be captured and related to one another in both machine and human readable formats. This removes ambiguities over the meaning of these terms, thus simplifying comparisons across different resources. We continually try to identify areas where adherence to FAIR principles with respect to Pfam data could be improved. As such, we have adopted the use of the Sequence Ontology (SO) [http://www.sequenceontology.org/] to assign a SO identifier to each of the six different entry types in Pfam (see Table 1). These SO terms are now included in both the Pfam website and flatfiles.

**Table 1.** SO terms that have been added in Pfam 32.0 for each Pfam type

Type	SO id	SO name
Coiled-coil	SO:0001080	coiled_coil
Disordered	SO:0100003	intrinsically_unstructured_polypeptide_region
Domain	SO:0000417	polypeptide_domain
Family	SO:0100021	polypeptide_conserved_region
Motif	SO:0001067	polypeptide_motif
Repeat	SO:0001068	polypeptide_repeat

Further to the addition of SO identifiers to our types, we have reviewed type definitions for 400 Pfam entries between releases 29.0 and 32.0. This has primarily focused on cases with long profile HMMs (lengths over >300) that are not of type *family*, those with known structures, or of inconsistent types within clans. During this survey, the single largest inconsistency within clans was found amongst those Pfam clans representing repeats.

## IMPROVING REPEAT DEFINITIONS IN Pfam

The identification and detection of tandem repeat (TR) regions by sequence analysis is a challenging task for many reasons, including their relatively short size (typically <60 aa), the degeneracy of the repeat at sequence level within a single protein, and the difficulty in accurately identifying the boundaries of each TR unit. Furthermore, repeats often correspond to areas of low compositional complexity, e.g. disordered regions, such that separating the two different signals can be challenging. Nevertheless, perfect repeats in engineered sequences do exist and ironically, often correlate with a tendency to be unstructured (18). On the other hand, naturally occurring proteins are characterised by low sequence conservation between repeating units. Consequently, different strategies are applied for identifying and classifying them, which are described in more detail in the following advanced online training course (https://www.ebi.ac.uk/training/online/course/repeats-pfam).

One approach is based on sequence homology detection: since sequence is conserved among evolutionarily related proteins, it is possible to use profile HMMs to identify repeats. This has been applied in Pfam TR model building with different representations of the repeat units, such as:

- Building a profile HMM that represents the individual unit (the desired approach), such as the case described below for HEAT- like repeats.
- Including multiple consecutive repeat units’ sequences in the seed alignment, since longer profile HMMs provide a better signal for the detection of true members. This strategy has been widely applied in cases like Leucine Rich Repeats or Ankyrin repeats (see Pfam: CL0022 and CL0465).
- Creating a single profile that models the entire TR region.

The latter two strategies, although often more sensitive, can be less desirable because they lead to partial overlaps between detected repeats and/or the omission of some units. No single approach ensures the proper identification of the correct periodicity of the repeat nor the representation of the TR in its entirety. Indeed, the tendency of repeated sequences to diverge is especially true for flanking units, meaning that even long models representing the entire TR often fail to completely represent the TR region in Pfam.

In order to improve our repeat definitions in Pfam, we have established a collaboration with RepeatsDB (19), a database focused on defining repeats in known structures. The strategy deployed by RepeatsDB is based on careful analysis of repeat structures where curators discern the start and the end of each repeat unit, as well as the number of repeats per structure. In conjunction with RepeatsDB, we can combine information available on repeats to consolidate our profile HMM models with structure information. This strategy is advantageous in numerous ways, including the identification of repeats not previously included in Pfam. Thus far, 39 new entries have been deposited in Pfam by the RepeatsDB curators. The RepeatsDB team are contributing to the revisions of Pfam repeat models to refine the boundaries of the repeat. For example, the Tal effector repeat (Pfam: PF03377) was altered to better agree with the known structural repeat. There is also an ongoing effort in grouping specific models representing the same structural unit within the same clan. This can be illustrated by the HEAT repeats, including the Importin HEAT-like repeat, which have six different and specific entries reflecting their high sequence diversity. The overall coverage can be increased by grouping the sequence models.

## UPDATING ANNOTATION FOR DOMAINS OF UNKNOWN FUNCTION

If possible, whenever a Pfam entry is created, a meaningful name based on its function is assigned, i.e. Pfam identifier). Where there is little or no functional information available, we call the entry a DUF and assign a sequential number, e.g. DUF100. The entry is re-annotated and renamed once the link between experimental evidence indicating a function has been made for the DUF.

Between releases 29.0 and 32.0, we added annotation and updated the names of 272 DUF entries. The functional annotation for these entries is derived from a variety of sources, but primarily through the discovery of references of Pfam accessions and/or DUFs in literature searches, updates provided by the scientific community via our helpdesk (see below), and via comparisons to other databases. As previously mentioned, ECOD was used to update DUF5328 to Cas6b_C (Pfam: PF17262) and was also used to rename DUF2945 to Hva1_TUDOR (Pfam: PF11160), after noting that the hypervirulence-associated protein 1 contains this domain. Furthermore, this entry was also added to the Tudor domain clan (CL0049). The majority of annotation updates have nonetheless come from InterPro curators. Pfam is part of the InterPro consortium, an amalgamation of 14 different expert databases (20), which present a single, unified view of regions of functional importance in protein sequences. InterPro curators integrate related entries from different member databases into InterPro entries, in order to provide a single resource with comprehensive coverage and a range of functional annotations. Furthermore, InterPro is updated every month to the latest version of UniProtKB. Consequently, once Pfam entries are integrated, InterPro curators are capable of identifying functions for DUFs based on the most recent sequence database update cycle, combined with the model information from the other member databases. The information is then returned to Pfam, enabling the entries in both resources to be consistently updated, ensuring efficient use of limited curation resources.

## CREDITING AUTHORSHIP OF Pfam ENTRIES

While much of the curation is performed by Pfam curators, we also rely on contributions from the wider scientific community. We frequently receive major annotation updates and suggested new Pfam entries from users of this resource, see https://www.ebi.ac.uk/training/online/course/pfam-database-creating-protein-families for more details on creating Pfam style entries. Others within the scientific community regularly provide bulk submissions for both existing and new entries. To recognize the contribution made by all Pfam curators and encourage more users to submit their work in a free and open format, we have extended our author lines (‘#=GF AU’ prefixed lines in the STOCKHOLM formatted flatfiles) to include, where possible, the authors’ Open Researcher and Contributor identifiers, termed ORCID (https://orcid.org/). ORCIDs provide persistent identifiers that link researchers to their various scholarly activities. To date, these identifiers have primarily been used to link researchers to their publications, but the ORCID system has been extended to allow the tagging of other works, such as peer review and contributions to databases. Of the 313 unique Pfam authors, just over half (161) have an associated ORCID. However, 97% of Pfam entries have at least one author with an ORCID. To prevent entries being claimed against the wrong profile, we request authors to supply their ORCID IDs along with their contributions which are then included in our database. Once a link is established between an ORCID and Pfam entry, the researcher can link their Pfam contributions to their ORCID profile by claiming them via the EBI-Search system (21) (see the ‘Authorship’ page in the help section of the Pfam website https://pfam.xfam.org/help for more details on claiming entries). The Pfam website has been updated to provide a link to each author's ORCID profile. Furthermore, these ORCIDs are also included in the flatfiles available via the FTP site, where each author is now displayed on a separate line, rather than the concatenated list previously provided. This is an important step in recognizing the substantial volume of curation by relatively few scientists over the past 20 years of Pfam’s existence.

## DISCUSSION

While Pfam constantly endeavours to remain comprehensive, there exists nearly 25% of sequences in *pfamseq* that are pending annotations by Pfam. The addition of hundreds of new entries has made little impact on the fraction covered, primarily due to the expansion of the sequence databases, thereby demonstrating the continual need for curation of new Pfam entries. We hope that the increased recognition that our contributors receive will encourage greater participation from the scientific community. In addition to the net gain of 1,647 entries, there has been significant focus on increased residue coverage and improving the boundaries and sensitivity of existing entries.

Despite our continual efforts to rename DUFs based on functional information found in both the scientific literature and other protein family databases (22), the fraction of Pfam entries with no known function has increased over the past decade. The current Pfam 32.0 release contains 3,961 DUFs (corresponding to 22% of all entries), in addition to entries that have not been labelled as DUFs, since they are referred to by common names derived from literature [e.g. HCMV_UL124 (Pfam: PF17609) is a family of viral membrane glycoproteins of unknown function]. We estimate that over a quarter of Pfam entries lack an experimentally validated function, highlighting the desperate need for more high-throughput functional screening of proteins. In the meantime, hierarchical classifications of proteins such as Pfam clans, have expanded substantially over the past 5 years, with 74% of all Pfam regions now belonging to a clan. Linking DUFs to functionally characterized members within a clan can provide important insights into the potential role of these functionally uncharacterized sequences. This, coupled with new approaches to functional prediction of protein family function, will be essential to bridge the experimental gaps.

## References

[1] Sonnhammer E.L., Eddy S.R., Durbin R. Pfam: a comprehensive database of protein domain families based on seed alignments. Proteins. 1997; 28:405–420. 9223186 10.1002/(sici)1097-0134(199707)28:3<405::aid-prot10>3.0.co;2-l

[2] Finn R.D., Coggill P., Eberhardt R.Y., Eddy S.R., Mistry J., Mitchell A.L., Potter S.C., Punta M., Qureshi M., Sangrador-Vegas A. The Pfam protein families database: towards a more sustainable future. Nucleic Acids Res. 2016; 44:D279–D285. 26673716 10.1093/nar/gkv1344 PMC4702930

[3] Sonnhammer E.L., Eddy S.R., Birney E., Bateman A., Durbin R. Pfam: multiple sequence alignments and HMM-profiles of protein domains. Nucleic Acids Res. 1998; 26:320–322. 9399864 10.1093/nar/26.1.320 PMC147209

[4] Eddy S.R. Accelerated profile HMM searches. PLoS Comput. Biol. 2011; 7:e1002195. 22039361 10.1371/journal.pcbi.1002195 PMC3197634

[5] The UniProt Consortium UniProt: the universal protein knowledgebase. Nucleic Acids Res. 2018; 45:D158–D169. 10.1093/nar/gkw1099 PMC5210571 27899622

[6] Sammut S.J., Finn R.D., Bateman A. Pfam 10 years on: 10,000 families and still growing. Brief. Bioinform. 2008; 9:210–219. 18344544 10.1093/bib/bbn010

[7] Rawlings N.D., Barrett A.J., Thomas P.D., Huang X., Bateman A., Finn R.D. The MEROPS database of proteolytic enzymes, their substrates and inhibitors in 2017 and a comparison with peptidases in the PANTHER database. Nucleic Acids Res. 2018; 46:D624–D632. 29145643 10.1093/nar/gkx1134 PMC5753285

[8] Finn R.D., Mistry J., Schuster-Böckler B., Griffiths-Jones S., Hollich V., Lassmann T., Moxon S., Marshall M., Khanna A., Durbin R. Pfam: clans, web tools and services. Nucleic Acids Res. 2006; 34:D247–D251. 16381856 10.1093/nar/gkj149 PMC1347511

[9] Bursteinas B., Britto R., Bely B., Auchincloss A., Rivoire C., Redaschi N., O’Donovan C., Martin M.-J. Minimizing proteome redundancy in the UniProt Knowledgebase. Database (Oxford). 2016; 2016:baw139. 28025334 10.1093/database/baw139 PMC5199198

[10] Dawson N.L., Lewis T.E., Das S., Lees J.G., Lee D., Ashford P., Orengo C.A., Sillitoe I. CATH: an expanded resource to predict protein function through structure and sequence. Nucleic Acids Res. 2017; 45:D289–D295. 27899584 10.1093/nar/gkw1098 PMC5210570

[11] Andreeva A., Howorth D., Chandonia J.-M., Brenner S.E., Hubbard T.J.P., Chothia C., Murzin A.G. Data growth and its impact on the SCOP database: new developments. Nucleic Acids Res. 2008; 36:D419–D425. 18000004 10.1093/nar/gkm993 PMC2238974

[12] Bateman A., Finn R.D. SCOOP: a simple method for identification of novel protein superfamily relationships. Bioinformatics. 2007; 23:809–814. 17277330 10.1093/bioinformatics/btm034 PMC2603044

[13] Cheng H., Schaeffer R.D., Liao Y., Kinch L.N., Pei J., Shi S., Kim B.-H., Grishin N.V. ECOD: an evolutionary classification of protein domains. PLoS Comput. Biol. 2014; 10:e1003926. 25474468 10.1371/journal.pcbi.1003926 PMC4256011

[14] Schaeffer R.D., Liao Y., Cheng H., Grishin N.V. ECOD: new developments in the evolutionary classification of domains. Nucleic Acids Res. 2017; 45:D296–D302. 27899594 10.1093/nar/gkw1137 PMC5210594

[15] Berman H., Henrick K., Nakamura H. Announcing the worldwide Protein Data Bank. Nat. Struct. Biol. 2003; 10:980–980. 14634627 10.1038/nsb1203-980

[16] Berman H., Henrick K., Nakamura H., Markley J.L. The worldwide Protein Data Bank (wwPDB): ensuring a single, uniform archive of PDB data. Nucleic Acids Res. 2007; 35:D301–D303. 17142228 10.1093/nar/gkl971 PMC1669775

[17] Wilkinson M.D., Dumontier M., Aalbersberg I.J.J., Appleton G., Axton M., Baak A., Blomberg N., Boiten J.-W., da Silva Santos L.B., Bourne P.E. The FAIR Guiding Principles for scientific data management and stewardship. Sci. Data. 2016; 3:160018. 26978244 10.1038/sdata.2016.18 PMC4792175

[18] Jorda J., Xue B., Uversky V.N., Kajava A.V. Protein tandem repeats - the more perfect, the less structured. FEBS J. 2010; 277:2673–2682. 20553501 10.1111/j.1742-464X.2010.07684.x PMC2928880

[19] Paladin L., Hirsh L., Piovesan D., Andrade-Navarro M.A., Kajava A.V., Tosatto S.C.E. RepeatsDB 2.0: improved annotation, classification, search and visualization of repeat protein structures. Nucleic Acids Res. 2017; 45:3613–3613. 27928056 10.1093/nar/gkw1268 PMC5389603

[20] Finn R.D., Attwood T.K., Babbitt P.C., Bateman A., Bork P., Bridge A.J., Chang H.-Y., Dosztányi Z., El-Gebali S., Fraser M. InterPro in 2017-beyond protein family and domain annotations. Nucleic Acids Res. 2017; 45:D190–D199. 27899635 10.1093/nar/gkw1107 PMC5210578

[21] Park Y.M., Squizzato S., Buso N., Gur T., Lopez R. The EBI search engine: EBI search as a service-making biological data accessible for all. Nucleic Acids Res. 2017; 45:W545–W549. 28472374 10.1093/nar/gkx359 PMC5570174

[22] Bateman A., Coggill P., Finn R.D. DUFs: families in search of function. Acta Crystallogr. Sect. F Struct. Biol. Cryst. Commun. 2010; 66:1148–1152. 10.1107/S1744309110001685 PMC2954198 20944204

